# Assessing ventilatory efficiency at rest in asthma: A longitudinal comparison with healthy subjects

**DOI:** 10.14814/phy2.70490

**Published:** 2025-07-30

**Authors:** Monique van Oosten, Arni Johnsen, Bjorn Magnusson, Marta Gudjonsdottir

**Affiliations:** ^1^ Physiotherapy Monique slf Mosfellsbaer Iceland; ^2^ Faculty of Medicine University of Iceland Reykjavik Iceland; ^3^ Department of Internal Medicine The South Coast Health Institution of Iceland Selfoss Iceland; ^4^ Cardiopulmonary Laboratory Reykjalundur Rehabilitation Centre Mosfellsbaer Iceland

**Keywords:** asthma control test, breath‐holding time, breathing pattern, dysfunctional breathing, ventilatory efficiency

## Abstract

Dysfunctional breathing, characterized by inefficient ventilation, is common among asthmatic patients. It is mainly identified using questionnaires and by observing breathing patterns, but the actual efficiency of ventilation is rarely evaluated. This study aimed to compare ventilatory efficiency at rest between asthmatic patients and healthy subjects, and to assess the stability of these parameters over 1 year. Ventilation (*V*_E_), respiratory rate (RR), tidal volume (V_T_), carbon dioxide output (*V*CO_2_) and end‐tidal partial pressure (P_ET_CO_2_), spirometry, and breath‐holding time (BHT) were measured at baseline (M1) and 17–51 weeks later (M2). The ventilatory efficiency (*V*_E_/*V*CO_2_) and breathing pattern (RR/V_T_) were calculated. Patients took the Asthma Control Test questionnaire (ACT). A mixed‐design ANOVA at M1 showed that 30 not well‐controlled asthmatic patients according to ACT (77% females, 7 with airway obstruction), and 23 matched healthy subjects had similar breathing parameters, except for BHT (*p* < 0.02) and P_ET_CO_2_ (*p* = 0.06). Only BHT changed, that is, it increased from M1 to M2 in the groups and remained shorter among the patients. Despite a shorter BHT among the patients, indicating a heightened perceptual sensitivity for dyspnoea, the ventilatory efficiency and breathing pattern at rest were similar and remained stable for 1 year in both groups.

## 1 INTRODUCTION

Asthma affects over 260 million people globally (Vos et al., 2020), yet its true prevalence remains uncertain due to diagnostic challenges (Aaron et al., 2018). It is a complex, heterogeneous disease characterized by airway inflammation, variable expiratory airflow, and fluctuating respiratory symptoms (Ginareport, 2025). Asthma often coexists with comorbidities, such as dysfunctional breathing (DB), also referred to as inefficient ventilation (Boulding et al., 2016).

Efficient ventilation occurs when ventilation (*V*_E_), which is composed of different combinations of respiratory rate (RR) and tidal volume (V_T_), is tightly controlled to maintain the partial pressure of carbon dioxide in arterial blood (PaCO_2_) at a set point as part of acid–base homeostasis. Therefore, ventilation must match the organism's metabolism, with minimal respiratory work for each breath (Mortola, 2019).

DB, on the other hand, is characterized by recurrent or chronic inefficient ventilation, leading to increased variability of breathing patterns (Ruane et al., 2024) with abnormally high respiratory work. Inefficient ventilation is indicated by high *V*_E_/carbon dioxide output (*V*CO_2_) and RR/V_T_ (Mortola, 2019; Wasserman et al., 2005). DB can lead to features such as dyspnoea, as well as other non‐respiratory and respiratory symptoms, and may result in either iso‐capnic hyperpnoea or hyperventilation, leading to hypocapnia (Gardner, 1996). It can occur independently, without underlying pulmonary pathology, or in conjunction with respiratory diseases such as asthma (Vidotto et al., 2019; Vlemincx, 2023).

Hyperventilation is clinically relevant in asthma, as hypocapnia may contribute to the pathophysiology of bronchoconstriction (Van den Elshout, 1991; Vasileiadis et al., 2019) and reinforce a cycle of inefficient ventilation, breathing vigilance, and anxiety (Vlemincx, 2023). Up to 94% of individuals with hyperventilation symptoms have asthma (Demeter & Cordasco, 1986; Saisch et al., 1996). Approximately 40% of stable and 74% of acute asthmatic patients show hyperventilation on blood gas analysis (Deenstra et al., 2022; McFadden Jr. & Lyons, 1968; Osborne et al., 2000; Tai & Read, 1967). Asthmatic patients may exhibit DB, that is, inefficient ventilation, without hyperventilation due to disordered breathing patterns (Todd et al., 2018), although the lack of a standardized definition complicates diagnosis and comparison across studies (Agache et al., 2012; Ruane et al., 2024). DB is most commonly assessed using subjective tools such as the Nijmegen Questionnaire (NQ), originally designed to detect hyperventilation in healthy adults (van Dixhoorn & Duivenvoorden, 1985; van Dixhoorn & Folgering, 2015). Although widely used, the NQ may overestimate DB (Agache et al., 2012) and fail to capture irregular breathing patterns (Todd et al., 2018). Given the complexity of breathing, questionnaires alone are insufficient for an accurate diagnosis of DB in asthma (Boulding et al., 2016; van Dixhoorn & Folgering, 2015). Additional tools such as plethysmography (Parreira et al., 2012; Sakkatos et al., 2021) and the Breathing Pattern Assessment Tool (Todd et al., 2018) are useful in identifying breathing pattern disorders in asthma; however, they do not assess hyperventilation or ventilatory efficiency (Deenstra et al., 2022; Hul et al., 2018). A multi‐component assessment is recommended to improve DB detection (van Dixhoorn & Folgering, 2015), combining subjective tools with objective ventilatory measures, such as PaCO_2_, *V*_E_/*V*CO_2_, and RR/V_T_, in resting conditions to help identify and explain DB patterns (Boulding et al., 2016; Habedank et al., 1998; Wasserman et al., 2005). Dyspnoea is the most commonly reported symptom in both asthma and DB (Vidotto et al., 2019), typically assessed using questionnaires (Crisafulli & Clini, 2010). Breath‐holding time (BHT) has also been used as an effective method of triggering dyspnoeic sensations and a simple, indirect measure of respiratory chemosensitivity to CO_2_. Shorter durations indicate heightened respiratory chemosensitivity to CO_2_ (Nishino, 2009; Nishino et al., 1996), which may also suggest a tendency toward hyperventilation (Gardner, 1996). However, inconsistencies in BHT measurement protocols limit comparability across studies (Courtney & Cohen, 2008; Jack et al., 2004; Messineo et al., 2021; Nishino, 2009; Nishino et al., 1996; Vigran et al., 2019). As DB is associated with not well‐controlled asthma (Sedeh et al., 2020), it should be assessed independently to improve disease management (Agache et al., 2012). Current guidelines recommend breathing training to reduce symptoms and improve quality of life (Ginareport, 2025), yet its impact on *V*_E_/*V*CO_2_ and RR/V_T_ as well as PaCO_2_ as measures of ventilatory efficiency remains largely unknown. Few controlled studies have objectively compared ventilation in asthmatic patients and healthy subjects using parameters such as PaCO_2_ and P_ET_CO_2_ (Bowler et al., 1998; Hormbrey et al., 1988; Kassabian et al., 1982; Osborne et al., 2000; Tobin et al., 1983). These studies consistently report increased ventilation in asthma. However, none of these studies evaluated the temporal stability of breathing parameters. For assessing DB in asthma during interventions like breathing training, reliable interpretation of changes requires stable measurements over time. Without this, it is unclear whether observed differences reflect true intervention effects or result from measurement variability. This study aimed to determine whether asthmatic patients exhibit impaired ventilatory efficiency, indicated by elevated *V*_E_/*V*CO_2_ and RR/V_T_ and reduced P_ET_CO_2_, compared to healthy subjects. Additionally, respiratory chemosensitivity to CO_2_ was assessed using BHT. The temporal stability of these parameters was evaluated under controlled resting conditions to assess their reliability for monitoring the effects of asthma interventions, such as breathing training.

## 2 MATERIALS AND METHODS

### 2.1 Participants

Asthmatic patients, ≥18 years old, were recruited by advertising at medical centers and on the homepage of The Icelandic Asthma‐ and Allergy Association. In addition, nurses and physicians treating asthmatic patients were informed about the study. All patients had physician‐diagnosed asthma and had inhaled short‐acting beta2‐agonist (SABA) once or more often in the 4 weeks before the study. Healthy subjects were friends and relatives of the patients and the researchers. They were matched for gender, age, and Body Mass Index (BMI) and had no history of pulmonary diseases or other conditions requiring medical therapy. All participants signed a consent form approved by The National Bioethics Committee of Iceland (VSNb2012010044/03.7).

### 2.2 Study design and data collection

This was a prospective, observational study. The participants were measured twice. The first measurement (M1) was carried out at baseline and the second measurement (M2) was performed 17 to 51 weeks later. The participants attended M2 at a time convenient to them, and the variable time intervals were evaluated to see if they would affect the results. The participants were instructed to maintain their usual lifestyle between measurements, including physical activity and their asthma management.

The time since the initial diagnosis, history of comorbidities, use of inhaled corticosteroids, oral corticosteroids, long‐acting beta2‐agonists, and short‐acting beta2‐agonists (SABA), and smoking history of all participants was recorded.

To minimize any provocation of breathing parameters and BHT, measurements were conducted under controlled resting conditions. The participants were instructed to refrain from consuming food, alcohol, caffeine, or asthma medication, as well as engaging in physical activity, for at least 8 h before testing. The measurements were taken before noon, during the weekend, in the following sequence: weight and height, resting ventilation and metabolism, BHT, and finally, spirometry. After completing these assessments, the asthmatic patients filled out the Asthma Control Test (ACT) questionnaire.

Ventilation (*V*_E_), respiratory rate (RR), tidal volume (V_T_), carbon dioxide output (*V*CO_2_) as a measure of metabolism and end‐tidal carbon dioxide partial pressure (P_ET_CO_2_) were analyzed breath by breath with a metabolic cart device (Vmax Encore 29, Sensormedics, CA, USA) and a facemask (7900 Hans Rudolph Inc, KC, MO, USA). Ventilation and metabolism were measured while participants sat upright and listened to the same relaxing audio recording for 15 min. The average of the last 4 min of measurements was used for analysis. Before each measurement, the gas and flow analyzers were calibrated. Spirometry was performed using the same device equipped with a mouthpiece and nose clip, measuring forced vital capacity (FVC) and forced expiratory volume in 1 s (FEV_1_), according to guidelines (Miller et al., 2005). The spirometry results were presented as absolute values and percentages of predicted values (Hankinson et al., 1999). The ventilatory equivalent for CO_2_ (*V*_E_/*V*CO_2_) as a measure of ventilatory efficiency and breathing pattern (RR/V_T_) were calculated. BMI was calculated by dividing the whole‐body weight in kilograms by height in meters squared (kg/m^2^).

BHT in this study was used to evaluate respiratory chemosensitivity to CO_2_. To determine the BHT, the participants were assisted to sit straight, relax, and breathe normally through their nose if possible. They were asked to hold their breath after a normal exhalation, pinch their nose gently until they experienced the first urge to breathe again, and then continue to breathe normally through the nose. The measurement procedure was considered correct if the breathing pattern remained consistent before and after the breath‐holding (Nishino, 2009; Nishino et al., 1996). BHT was measured using a stopwatch and was repeated three times, with a time interval between measurements long enough for the breathing pattern to have become stable again or for at least 1 min. The mean of the three measurements was used for data analysis.

The patients filled out the ACT questionnaire, a reliable, guideline‐supported, five‐item measure of asthma control sensitive to change (Michael Schatz et al., 2006). ACT scores range from 5 to 25. Scores of 20–25 signify well‐controlled asthma, 16–19 represent not well‐controlled asthma, and 5–15 signify very poorly controlled asthma. The minimally important change in ACT scores is three points (Schatz et al., 2009).

### 2.3 Statistical analysis

A power analysis was conducted to determine the sample size, assuming an alpha level of significance of 5% and a test power of 90% (*n* = 20 for each group). Data were analyzed using R version 3.6.2 (R Core Team, 2019). Categorical variables were presented as numbers (percentages). The Shapiro–Wilk test was used to test the normality of data. Continuous variables were presented as mean ± standard deviation (SD) if normally distributed and as median [1st quartile, 3rd quartile] if not.

A mixed‐design Analysis of Variance (ANOVA) analyzed differences in continuous variables within and between groups. No extreme outliers were identified in the data. Levene's test for equality of variances was used to test for homogeneity of variance between groups, and Box's M test was used to test for homogeneity of covariance. A mixed‐design ANOVA on the trimmed means was used for variables that did not fulfill assumptions of normality, homogeneity of variance, or homogeneity of covariance. Differences in categorical variables were analyzed with a chi‐squared test. The correlation between continuous variables was assessed with Spearman's rank correlation coefficient. Univariate linear regression was used to determine if the time between measurements affected the change in measured variables, using the time between measurements as the explanatory variable and the change in any measured variable as the dependent variable. The level of statistical significance was defined as *p* < 0.05 for all statistical tests, except for Box's M test, where it was defined as *p* < 0.001.

## 3 RESULTS

Fifty‐nine participants were recruited. Six asthmatic patients voluntarily withdrew their participation before the M2 measurement and are therefore not included in the results. The characteristics of the participants at M1 are presented in Table 1. More than half of the patients had a history of childhood asthma. The average time since initial diagnosis was 45.7 years for these patients and 31.2 years across the entire cohort. Asthma was, on average, not well‐controlled, as indicated by mean ACT scores of 16.2 ± 4.6 at M1 and 18.5 ± 5.0 at M2 (*p* < 0.05). Seven of the 30 asthmatic patients were obstructed (FEV_1_/FVC < 0.70), of which three were mildly obstructed (FEV_1_% predicted >80%), three moderately (FEV_1_% predicted 60%–80%) and one severely (FEV_1_% predicted = 35%), and one in the healthy subjects group appeared to be mildly obstructed.

**TABLE 1:** Characteristics of the participants at baseline (M1).

	Patients, *n* = 30	Controls, *n* = 23	*p*‐value
Age, years	45.0 ± 14.2	44.2 ± 15.3	0.846
Female, *n* (%)	23 (76)	17 (74)	1.0
Weight, kg	78.8 ± 15.7	79.3 ± 13.0	0.911
Height, cm	169 ± 8	173 ± 10	0.108
BMI, kg/m^2^	27.7 ± 4.9	26.6 ± 4.8	0.393
Smokers, *n*	3	3	
Ex‐smokers, *n*	1	0	
Medication			
SABA 1–3/week, *n* (%)	12 (40)	0	
SABA 2–3/day, *n* (%)	18 (60)	0	
ICS and/or LABA *n* (%)	24 (80)	0	
Comorbidity			
None, *n* (%)	6 (20)	23 (100)	
Allergy and/or rhinitis, *n* (%)	6 (53)		
Fibromyalgia, *n* (%)	6 (20)		
GERD, *n* (%)	6 (20)		
Other^a^, *n* (%)	12 (40)		

*Note*: Data are presented as mean ± SD or as number, *n*, and percentage (%).

Abbreviations: BMI, Body Mass Index; GERD, gastroesophageal reflux disease; ICS, inhaled corticosteroid; LABA, inhaled long‐acting beta2‐agonist; *n*, number; percentage (%); SABA, inhaled short‐acting beta2‐agonist.

^a^ Depression (*n* = 3); obesity (*n* = 2), Addison's disease (*n* = 1); hypertension (*n* = 2); Crohn's disease (*n* = 1); diabetes type II (*n* = 1); hyperthyroidism (*n* = 1); hyperlipidemia (*n* = 1).

The time interval between the M1 and M2 measurements ranged from 16.7 to 50.9 weeks. The interval was 25.2 ± 4.3 weeks for asthmatic patients and 33.4 ± 7.8 weeks for healthy subjects (*p* < 0.001). The average BMI did not change over time; at M2, it was 27.7 ± 5.0 kg/m^2^ in the asthmatic group and 26.5 ± 5.0 kg/m^2^ in the control group (for change over time, *p* = 0.904).

Measurements of spirometry, breath‐holding time, resting ventilation, and metabolism measured at M1 and at M2 are shown in Table 2. Pulmonary function was within the normal range for both groups, except for FEF_25‐75%_ in the asthmatic patients, and none of these parameters changed over time. BHT was shorter in the asthmatic patients but increased over time in both groups in a similar manner and remained shorter in the asthmatic group. The P_ET_CO_2_ trended to be significantly lower in the asthmatic patients (*p* = 0.06) and did not change over time. The various time intervals between measurements did not significantly affect any of the abovementioned parameters.

**TABLE 2:** Measurements of spirometry, breath‐holding time, resting ventilation, and metabolism measured at baseline (M1) and at the second measurement (M2).

	Patients *n* = 30		Controls *n* = 23		*p‐*value^a^	*p*‐value^b^	*p*‐value^c^
	M1	M2	M1	M2			
FVC, % pred	108 [99, 123]	107 [100,126]	115 [102,125]	112 [101, 126]	0.656	0.467	0.787
FEV_1_, % pred	98 [88, 108]	98 [94, 114]	109 [100, 114]	104 [100, 116]	0.093	0.704	0.151
FEV_1_/FVC	0.76 [0.70, 0.83]	0.78 [0.72, 0.83]	0.82 [0.76, 0.84]	0.79 [0.76, 0.84]	0.084	0.484	0.068
FEF_25–75%_, % pred	66 [50, 95]	74 [62, 93]	93 [74,110]	94 [74,109]	0.0005	0.624	0.405
BHT, seconds	12.7 [9.2, 17.2]	14.4 [10.7, 18.5]	17.2 [13.6, 23.3]	21.1 [13.5, 26.7]	0.011	0.031	0.994
*V*_E_, L/min	6.0 [5.6, 7.7]	6.4 [5.5, 7.6]	6.0 [5.5, 7.6]	6.3 [5.6, 7.3]	0.921	0.725	0.627
*V*_E_/*V*CO_2_	47.1 [42.5, 55.4]	43.8 [39.8, 52.9]	41.2 [39.3, 47.1]	42.1 [37.6, 47.8]	0.085	0.239	0.16
RR/V_T_	23.0 [15.1, 35.5]	21.6 [11.7, 28.6]	19.9 [11.7, 23.8]	16.0 [13.5, 25.9]	0.214	0.283	0.365
P_ET_CO_2_, mmHg	33.6 ± 3.41	33.1 ± 3.66	34.8 ± 2.8	35.0 ± 2.55	0.06	0.703	0.324

*Note*: Data are presented as mean ± SD or median [interquartile range].

Abbreviations: BHT, breath‐holding time; FEF_25‐75%_, forced expiratory flow rate between 25% and 75% of the forced vital capacity; FEV_1_, forced expired volume in 1 s; FEV_1_/FVC, ratio between FEV_1_ and FVC; FVC, forced vital capacity; *n*, number; P_ET_CO_2_, partial pressure of end‐tidal carbon dioxide; RR, respiratory rate; RR/V_T_, breathing pattern; *V*CO_2_, carbon dioxide output; *V*_E_, ventilation; *V*_E_/*V*CO_2_, ventilatory efficiency; V_T_, tidal volume.

^a^ Between group difference.

^b^ Within group difference over time.

^c^ Interaction of group by time [group × time].

**FIGURE 1:**
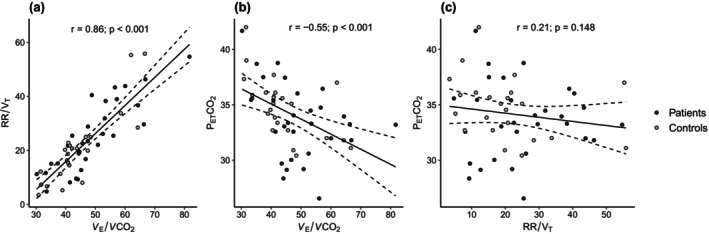
Correlations between ventilatory parameters in all participants at M1. Relationship between (a) breathing pattern (RR/V_T_) and ventilatory efficiency (*V*_E_/*V*CO_2_), (b) end‐tidal carbon dioxide (P_ET_CO_2_) and *V*_E_/*V*CO_2_ ratio, and (c) P_ET_CO_2_ and RR/V_T_. M1 = measurement at baseline; RR/V_T_ = ratio of respiratory rate and tidal volume (i.e., breathing pattern).

**FIGURE 2:**
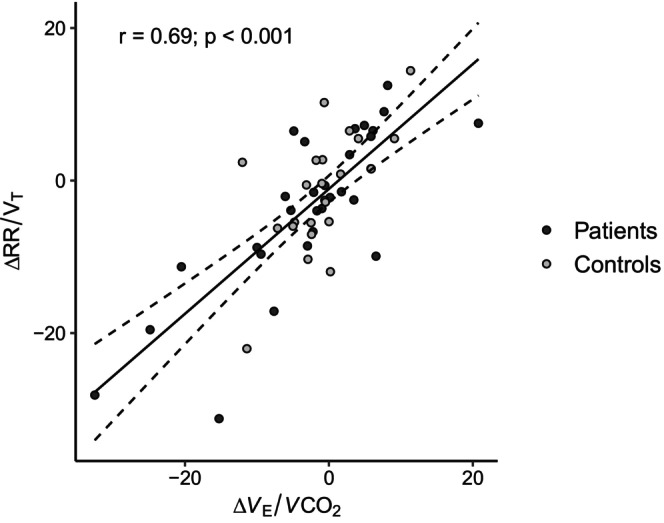
Relationship between changes of ventilatory efficiency (*V*_E_/*V*CO_2_) and breathing pattern (RR/V_T_) from M1 to M2 in all participants. M2 = second measurement; RR/V_T_ = ratio of respiratory rate and tidal volume (i.e., breathing pattern).

The linear relationships between the ventilatory efficiency (*V*_E_/*V*CO_2_), breathing pattern (RR/V_T_) and P_ET_CO_2_ in all participants at M1 are shown in Figure 1. The *V*_E_/*V*CO_2_ was positively correlated with the RR/V_T_ and negatively correlated with P_ET_CO_2_. No correlation was found between P_ET_CO_2_ and the RR/V_T_. BHT had a negative correlation with the *V*_E_/*V*CO_2_ (*r* = −0.50, *p* < 0.0001) and RR/V_T_ (*r* = −0.46, *p* < 0.001), but no correlation with P_ET_CO_2_ (*r* = 0.21, *p* = 0,127). In the asthmatic patients, the ACT questionnaire scores did not correlate with any breathing parameters nor BHT in M1. The relationship between changes from M1 to M2 in *V*_E_/*V*CO_2_ and RR/V_T_ in all participants also showed a positive correlation, as shown in Figure 2. Changes of P_ET_CO_2_ and *V*_E_/*V*CO_2_ from M1 to M2 were negatively correlated (*r* = 0.388, *p* = −0.0041), and there was no correlation between changes in P_ET_CO_2_ and RR/V_T_ (*r* = 0.03, *p* = 0.83). In the asthmatic patients, a negative correlation was found between changes in BHT and ACT questionnaire scores (*r* = −0.41, *p* < 0.05).

## 4 DISCUSSION

This study found that ventilatory efficiency and breathing patterns were similar between asthmatic patients and healthy subjects, although P_ET_CO_2_ tended to be lower in the asthma group. None of the measured parameters changed over time in either group. Breath‐holding time, on the other hand, was shorter among the patients, indicating higher respiratory chemosensitivity to CO_2_. Furthermore, breath‐holding time increased over time in both groups. Mean ACT scores indicated not well‐controlled asthma despite regular inhalation medication use, with a non‐clinically significant increase observed over time.

### 4.1 Methodology

To our knowledge, this is the first study to objectively assess ventilatory efficiency at rest by incorporating metabolic parameters, specifically *V*CO_2_, and comparing it between asthmatic patients and healthy subjects. Previous studies have examined ventilation in these groups, with or without measurements of PaCO_2_ and/or P_ET_CO_2_ (Bowler et al., 1998; Hormbrey et al., 1988; Kassabian et al., 1982; Osborne et al., 2000; Tobin et al., 1983). Two studies reported similar resting ventilation between asthmatic patients and healthy subjects but found lower PaCO_2_ and P_ET_CO_2_ in the asthmatic group (Bowler et al., 1998; Osborne et al., 2000). Two other studies (Hormbrey et al., 1988; Kassabian et al., 1982) observed hyperventilation in asthmatic patients, characterized by higher resting ventilation and lower P_ET_CO_2_ compared to healthy subjects; however, these studies did not account for metabolism. While reduced PaCO_2_ or P_ET_CO_2_ suggests hyperventilation, these measures alone do not fully capture other aspects of DB. By integrating metabolic assessment, our study provides a more comprehensive evaluation of ventilatory efficiency beyond hyperventilation alone (Habedank et al., 1998; Wasserman et al., 2005). Still, only a few studies have been conducted on resting ventilatory efficiency, and there is a need for larger studies to establish cut‐off scores on this subject (Habedank et al., 1998; Ries, 1987).

In the present study, validation regarding age (Han et al., 1997), gender (Thomas et al., 2005) and BMI (Fenger et al., 2014) was established by matching the two groups on these parameters. Except for Osborne (Osborne et al., 2000), this was not sufficiently controlled for in the other studies (Bowler et al., 1998; Hormbrey et al., 1988; Kassabian et al., 1982; Tobin et al., 1983). Two studies had no matching (Kassabian et al., 1982; Tobin et al., 1983), and the other two studies matched for only age and gender (Bowler et al., 1998; Hormbrey et al., 1988).

The contradictory results could also be due to methodological differences, considering that ventilation is a sensitive parameter influenced by various factors (Vlemincx, 2023). For example, withholding daily stress, food, physical activity, and medication (Haffner & Kendall, 1992) before the measurement is preferable as they can affect the results of breathing measurements. To minimize these influences, all participants in this study followed the same strict preparation protocol and were tested under controlled resting conditions, in the same laboratory with the same researchers, in the early morning and on weekends. Prior studies varied in their preparation protocols. Kassabian et al. (Kassabian et al., 1982) measured asthmatic patients during acute illness; Bowler et al. (1998) and Tobin et al. (1983) provided no preparation details, while Osborne et al. (Osborne et al., 2000) had participants abstain from medication and caffeine for 8 h prior to testing. Hombrey et al. (1988) offered the most standardized approach, controlling for food, caffeine, alcohol, and drug intake and measuring at 10 AM. Additionally, resting conditions during measurements, such as emotional state (Jerath et al., 2015; Melnychuk et al., 2018), position (Hodges et al., 2001), and the equipment used for measuring ventilation (Hirsch & Bishop, 1982), can influence breathing patterns. Position can affect the activation of the inspiratory muscles (Hodges et al., 2001) and anatomic dead space (Brewer et al., 2008). This study measured participants while they were listening to relaxing audio in an upright sitting position, similar to Hormbrey et al. (1988). By comparison, Tobin et al. (1983) measured participants in the supine position after 10 min of rest, whereas Bowler et al. (1998) provided no positional details. Other studies measured participants while they were sitting but did not specify whether they were in an upright position (Kassabian et al., 1982; Osborne et al., 2000). Finally, while most studies used a mouthpiece and nose clip, this study employed a face mask to measure breathing parameters, except for spirometry.

### 4.2 Respiratory chemosensitivity to CO2

In subjects with DB and asthma, breathlessness or dyspnoea is commonly observed (Boulding et al., 2016). Neural and emotional mechanisms influencing dyspnoea perception, coupled with bronchoconstriction, may drive physiologically inefficient and non‐adaptive breathing behaviors (Hayen et al., 2013), leading to increased breathing pattern variability, which is the primary mechanism of DB (Bokov et al., 2016). As the asthmatic patients in this study had a history of asthma lasting 31 years on average, DB can be an expected response in the long run to dyspnoea perception. This may have increased breathing vigilance, creating a vicious cycle of anxiety (Bokov et al., 2016; Vlemincx, 2023), increasing DB (Bokov et al., 2016). Bronchoconstriction can contribute to lung hyperinflation evidenced by increased RR/V_T_ and mean inspiratory flow or V_T_/TI ratios (Fukushi et al., 2021; Hayen et al., 2013; Kassabian et al., 1982; Tobin et al., 1983). As lung hyperinflation places a greater load on the inspiratory muscles, generating a heightened sense of respiratory effort, it can contribute to dyspnoea (Fukushi et al., 2021). Spirometry in this study revealed that 23 of the 30 asthmatic patients had a normal FEV_1_/FVC ratio (≥ 0.70); however, the asthmatic patients exhibited a lower FEF_25‐75%_ than the healthy subjects (*p* < 0.0005), indicating obstruction of the small airways. This was unlikely to induce hyperinflation in the asthmatic group during ventilation under controlled resting conditions, as done in our study, but could become evident during exercise (Pellegrino & Brusasco, 1997).

Voluntary breath‐holding is one of the most effective methods of triggering a dyspnoeic sensation and is the oldest and simplest method for studying respiratory chemosensitivity to CO_2_ (Nishino et al., 1996). Recent research concluded that breath‐holding times are a novel indicator for identifying high‐risk COVID patients (Messineo et al., 2021). However, researchers perform breath‐holding measurements differently, evaluating different issues, which makes it difficult to compare the results (Courtney & Cohen, 2008; Jack et al., 2004; Messineo et al., 2021; Nishino et al., 1996; Vigran et al., 2019).

According to the literature, the breath‐holding time after a normal exhalation maneuver can be divided into three phases: the first phase, a “no respiratory sensation period”, from functional residual capacity until the very first sensation of desire to breathe, BHT‐p1. This breath‐holding time provides information about the onset of the dyspnoea perception threshold in the absence of respiratory effort, leaving the respiratory chemosensitivity to CO_2_ to affect the set point (Nishino, 2009; Nishino et al., 1996). BHT‐p1 measured in this manner is minimally influenced by repeated trials (Nishino et al., 1996) and has been used in studies to evaluate the effect of breathing training (Grammatopoulou et al., 2017; Novozhilov, 2004; Vagedes et al., 2024). It could provide feedback on the risk of symptom recurrence and exacerbations, as well as progress monitoring, evidenced by longer BHT‐p1 durations, as respiratory chemosensitivity to CO_2_ decreases and CO_2_ levels rise during training. As the breath‐holding continues, the second phase of the breath‐holding occurs after this “no respiratory sensation period”, until involuntary chest movements are observed (Courtney & Cohen, 2008; Vigran et al., 2019). According to Vigran et al. (2019) this phase of breath‐holding can be influenced by psychological measures such as time manipulation. The third phase is from involuntary chest movements until the maximal tolerable breath‐holding is reached. A complex interaction of physiological and behavioral features determines the breakpoint of the tolerable limit (Courtney & Cohen, 2008; Nishino, 2009; Nishino et al., 1996; Vigran et al., 2019). It has been shown that repeated testing significantly lengthens this phase (Nishino et al., 1996). As different factors influence the latter two phases of breath‐holding, they are unreliable for assessing results in breathing training. They will probably show both longer and more variable outcomes of BHT, underestimating the respiratory chemosensitivity to CO_2_ (Nishino et al., 1996; Vigran et al., 2019).

In this study, where the duration of “no respiratory sensation” (BHT‐p1) was used, asthmatic patients had significantly shorter BHT‐p1 than healthy subjects at both M1 and M2. The inherent difference in experienced dyspnoea could explain the dissimilarity in BHT‐p1 measured in this study, indicating that the asthmatic patient's respiratory control centres were more sensitive. Therefore, it can be hypothesized that the patient's underlying central respiratory rhythm will respond more quickly when provoked by psychological, physiological, social, or environmental challenges. This could result in an exaggerated ventilatory response, triggering DB sooner than in healthy subjects (Duffin, 2005).

The possibility of hyperventilation in the asthmatic patients cannot be excluded, as P_ET_CO_2_ was marginally but not significantly lower (*p* = 0.06, Table 2). P_ET_CO_2_ is commonly used to estimate arterial CO_2_ partial pressure (PaCO_2_) in healthy individuals, though discrepancies may arise in asthma due to bronchoconstriction. Lower P_ET_CO_2_ values can underestimate true PaCO_2_ levels (Wasserman et al., 2005). However, Osborne et al. (Osborne et al., 2000) demonstrated that P_ET_CO_2_ reliably reflects PaCO_2_ even in mild‐to‐moderate asthma. In this study, although asthma was not well controlled according to the ACT questionnaire, spirometry showed mild‐to‐moderate obstruction in six of 30 asthmatic patients and severe obstruction in one. Thus, it is reasonable to assume that lower P_ET_CO_2_ reflects lower PaCO_2_, suggesting a tendency toward hyperventilation in the asthmatic patients. This finding aligns with previous studies: Osborne et al. (Osborne et al., 2000) reported hypocapnia in seven of 23 (30%) patients with mild‐to‐moderate stable asthma, while Tai & Read (1967) observed the same in 14 of 64 (22%) patients. Additionally, Deenstra et al. (2022) analyzed blood gases from 1,006 predominantly (60%) not well‐controlled asthmatic patients, revealing that 40% of all patients had hyperventilation.

### 4.3 Correlations of breathing parameters and BHT

Although the measured parameters were similar, except for BHT‐p1 in both groups, and did not change significantly from M1 to M2 in either group, except for BHT‐p1, they varied among the subjects in this study (Figure 1). The *V*_E_/*V*CO_2_ at M1 and changes in the *V*_E_/*V*CO_2_ from M1 to M2 were strongly associated with breathing pattern (RR/V_T_) and changes in breathing pattern and to a lesser extent with P_ET_CO_2_ at M1, but not with changes in P_ET_CO_2_. This could indicate that the *V*_E_/*V*CO_2_ was mainly affected by anatomical dead space ventilation through the breathing pattern, rather than by the CO_2_ set point. Iso‐capnic hyperpnoea, rather than hyperventilation, appears to be the primary reason for a higher *V*_E_/*V*CO_2_ in some patients and healthy subjects. Dysfunctional breathing was expected in both groups, particularly in the asthmatic group (Agache et al., 2012). In this study, conducted under controlled resting conditions, DB, as indicated by ventilatory efficiency (*V*_E_/*V*CO_2_) and breathing pattern (RR/V_T_), was similar in both groups. However, a shorter BHT‐p1 in the asthmatic group suggests increased respiratory chemosensitivity to CO_2_, indicating a higher response to sensations of dyspnoea. Our findings align with Deenstra et al. (2022), who suggested that the individual burden of asthma is more likely driven by the symptom‐amplifying effects of over‐breathing and resulting hypocapnia in chronic hyperventilation rather than bronchoconstriction severity.

### 4.4 Time interval and stability of measurements

To cover a timeframe in which post‐breathing training adaptations would appear, which can last from 12 to 52 weeks (Santino et al., 2020), the stability of the breathing parameters, BHT and ACT scores were evaluated by measuring twice (M1 and M2) over 1 year. The time interval between measurements was decided at the convenience of the participants. It ended with a variable time interval of 16.7–50.9 weeks and was shorter in the asthma group. The objective measures *V*_E_/*V*CO_2_, RR/V_T_, and P_ET_CO_2_ remained stable over the various time intervals between M1 and M2. If breathing training leads to improvements in ventilatory efficiency, and thereby mitigating aspects of DB, this would be expected to manifest as reductions in *V*_E_/*V*CO_2_ and RR/V_T_ and a possible increase in P_ET_CO_2_. Nevertheless, it cannot be excluded that these measures lack the sensitivity to detect such changes, particularly in the context of subtle adaptations over time. Evidence supporting the responsiveness of these indices to physiological intervention has been reported by Crabtree et al. (2025), where resting ventilation and metabolic rate were elevated following ingestion of a ketogenic promoting drink compared to placebo in 12 healthy individuals (Crabtree et al., 2025). These findings suggest that such measurements can reflect acute metabolic and ventilatory changes. In addition, prior work demonstrated a more favorable breathing pattern during exercise after physical training in two severely affected patient populations: those with heart failure and those with severe chronic obstructive pulmonary disease (Gudjonsdottir et al., 2020), further supporting the potential of these indices to reflect altered ventilatory mechanics under certain conditions.

However, the subjective measures, both ACT scores and BHT, improved between M1 and M2, despite instructions to maintain a habitual lifestyle and the absence of any formal intervention. The mean ACT scores remained in the 16–19 range at M2, classified as not well‐controlled asthma. Changes did not exceed the minimally important difference of three points (M. Schatz et al., 2009). To minimize learning effects, the BHT‐p1 was employed; still, this measure remains subject to the participant's perception of breathlessness. The requirement for consistent breathing patterns before and after breath‐holding does not preclude underestimation of BHT‐p1. This may contribute to the longer BHT‐p1 observed in both groups at M2, and it may also reflect a non‐specific adherence effect, suggesting the need for a habituating trial with the BHT procedure before data collection (Green et al., 2017). Notably, BHT‐p1 remained shorter in the asthmatic group at M2 compared to the healthy subjects, indicating an increased sensitivity to dyspnoea‐related sensations. These results align with the findings of Vagedes et al. (2024), who compared healthy subjects to asthmatic patients and reported shorter breath‐holding times in the asthmatic group at baseline (18.3 s vs. 13.8 s respectively). However, they observed no changes in breath‐holding time after 3 months in the asthmatic group; the healthy subjects were only measured once, and no follow‐up data were provided. Unlike the methodology in the present study, their BHT (control pause) was measured only once per session, with baseline and three‐month follow‐up measurements conducted at different times of day for feasibility and no information was provided about a habituation trial (Vagedes et al., 2024). The ACT similarly reflects subjective experience, incorporating both direct symptom perception and its interpretation in relation to asthma control and quality of life. Therefore, improvements in these measures may reflect, at least in part, research participation effects (McCambridge et al., 2014).

### 4.5 Strengths and limitations of the study

A strength of this study is that measurements were carefully controlled in optimal resting circumstances. This is crucial for evaluating breathing parameters and BHT, as ventilation, modulated by the autonomic nervous system, can be affected by multiple factors.

This study aimed to assess the stability of measures by measuring twice with different time intervals between M1 and M2, covering the time frame in which post‐breathing training adaptations would appear. The study's merit lies in its combination of ventilation and metabolic variables to accurately evaluate ventilatory efficiency (*V*_E_/*V*CO_2_). Since there is no cut‐off value for normal *V*_E_/*V*CO_2_, comparing the groups can help identify differences in ventilatory efficiency.

In addition, allowing comorbidities such as rhinitis, gastroesophageal reflux disease, and psychopathological comorbidities in the study makes it more representative of a “real‐life” asthma population (Papaioannou et al., 2015).

A limitation was the time elapsed since the last usage of short‐ and long‐acting inhaled asthma drugs. According to the guidelines of 2005 (Miller et al., 2005), the time interval for short‐acting drugs was 4 h and for long‐acting drugs was 12 h when the study was conducted. However, updated ERS/ATS spirometry guidelines, published after the initiation of data collection for this study, recommend a 24‐h interval (Graham et al., 2019).

Furthermore, it would have been more accurate to have measured PaCO_2_ (Wasserman et al., 2005), but this was beyond the scope of the study. Also, the NQ could have provided information about subjective symptoms associated with DB, particularly hyperventilation in the participants; however, this questionnaire was not available in the Icelandic language at the time this research was conducted. Finally, habituating the patients with the BHT‐p1 procedure could have led to more reliable results (Green et al., 2017).

## 5 CONCLUSION

This study, conducted under controlled resting conditions, demonstrated that ventilatory efficiency and breathing pattern were similar in asthmatic patients and healthy subjects, despite greater respiratory chemosensitivity to CO_2_ in the asthma group. Asthmatic patients also showed a tendency toward hyperventilation. Objectively measured breathing parameters remained stable over 1 year; however, the subjectively assessed BHT‐p1 increased in both groups, likely reflecting a research participation effect rather than a physiological change. The improvements in ATC observed in the asthma group did not exceed the minimally important difference, despite being statistically significant, highlighting the need to consider clinical relevance alongside statistical outcomes. These findings support the use of the present measurement approach for the objective evaluation of breathing therapies.

Future studies should explore ventilatory responses under conditions of mental stress or physical activity using objective methods. The elevated respiratory chemosensitivity to CO_2_ in asthma may exaggerate ventilatory responses and contribute to hyperventilation in such contexts.

## FUNDING STATEMENT

Funder One, the Icelandic Asthma and Allergy Association; Funder Two, the Icelandic Physiotherapy Association; Funder Three, the Research Fund of Reykjalundur Rehabilitation Centre; Funder Four, the Oddur Ólafsson Fund. These funding sources had no involvement in the study design. They have no known competing financial interests, nor are there any personal relationships that could have appeared to influence the work reported in this paper.

## CONFLICTS OF INTEREST

None of the authors have any conflicts of interest regarding the data presented or have published or submitted any related papers from the same study.

## ETHICS STATEMENT

The study was approved by the National Bioethics Committee of Iceland, registration number VSNb2012010044/03.7.

## CONSENT

All participants gave their consent for inclusion before they participated in the study: “I have read and understand the provided information and have had the opportunity to ask questions. I understand that my participation is voluntary and that I am free to withdraw at any time without giving a reason and without cost. I understand that I will be given a copy of this consent form.”

## Data Availability

The data that support the findings of this study are available from the corresponding author [MvO] upon reasonable request.
